# Mechanical Recycling of Ethylene-Vinyl Acetate/Carbon Nanotube Nanocomposites: Processing, Thermal, Rheological, Mechanical and Electrical Behavior

**DOI:** 10.3390/polym15030583

**Published:** 2023-01-23

**Authors:** Ionut-Laurentiu Sandu, Felicia Stan, Catalin Fetecau

**Affiliations:** Center of Excellence Polymer Processing, Dunarea de Jos University of Galati, 47 Domneasca, 800 008 Galati, Romania

**Keywords:** ethylene-vinyl acetate, carbon nanotubes, mechanical recycling, injection molding, thermal behavior, capillary rheometry, mechanical properties, electrical conductivity

## Abstract

Recycling polymer/carbon nanotube (CNT) nanocomposites is not well common, despite a growing interest in using polymer/carbon nanotube (CNT) nanocomposites in industrial applications. In this study, the influence of mechanical recycling on the thermal, rheological, mechanical and electrical behavior of ethylene-vinyl acetate (EVA)/CNT nanocomposites is investigated. EVA/CNT nanocomposite with different amounts of CNTs (1, 3 and 5 wt.%) was subjected to mechanical grinding and reprocessing by injection molding in a close-loop up to three cycles, and the changes induced by mechanical recycling were monitored by Differential Scanning Calorimetry (DSC), capillary rheology, scanning electron microscopy (SEM), electrical resistance and tensile tests. It was found that the EVA/CNT nanocomposites did not exhibit significant changes in thermal and flow behavior due to mechanical recycling and reprocessing. The recycled EVA/CNT nanocomposites retain close to 75% of the original elastic modulus after three recycling cycles and about 80–90% in the tensile strength, depending on the CNT loading. The electrical conductivity of the recycled nanocomposites was about one order of magnitude lower as compared with the virgin nanocomposites, spanning the insulating to semi-conducting range (10^−9^ S/m–10^−2^ S/m) depending on the CNT loading. With proper control of the injection molding temperature and CNT loading, a balance between the mechanical and electrical properties of the recycled EVA nanocomposites can be reached, showing a potential to be used in practical applications.

## 1. Introduction

The latest statistical results show that the production of plastic for 2021 is more than 390 million tones, from which only about 8% come from recyclable, the rest having raw material fossil-based (over 90%) and bio-based plastics (less than 2%) [1]. On the other hand, given the current socio-political conditions and consumer demands, it is estimated that global plastic use will triple by 2060 [2,3]. In Europe, despite the increasing plastic landfilling restrictions and the EU zero plastics to landfill goal by 2030 [4], a large amount of plastic waste is still produced from industrial, agricultural and household activities. It has been reported that about 60 million tonnes of plastics are produced in Europe every year, while only 30% of it is recycled [5,6]. Of all the plastic waste generated in the EU during 2020, more than 40% was used for energy recovery, 33% was sent to recycling, and 23% ended up in landfill or as litter in the natural environment [5,6]. Therefore, given the new economic, environmental and petroleum context, the scientific communities must increasingly deal with polymer recycling in order to increase the circularity of the plastics.

In the last years, nanotechnology has been assumed as one of the key technologies and substantial enhancements of various properties can be obtained by the addition of nanofillers [7,8]. Among nanofillers, carbon nanotubes (CNTs) have attracted much attention due to their excellent electrical, mechanical and thermal properties [9,10,11,12,13,14]. In the last years, CNTs have become more affordable due to the development of large-scale production capacity and are thus used as fillers in polymers [15].

Although the CNT production capacity is not yet comparable with that of, for example, carbon fibers, the global CNT market is expected to increase and reach a compound annual growth rate (CAGR) of 20% with a projected value of $6.03 Billion in 2027 [16] especially due to the high demand of the automotive, aeronautics, electrical, defense, oil, communications, and sports industry. On the other hand, the widespread use of CNT-based nanocomposites in consumer products calls for the assessment of the life cycle. It is expected that many consumer products have already reached end-of-life and ended up in the waste stream for waste incineration or landfills [17,18,19].

In general, post-industrial nanocomposite waste is recycled into new feedstocks for the plastic industry, and its economic value is recovered while the impact on the environment is minimized if not avoided. On the other hand, no public information exists on the amount of post-consumer polymer/CNT waste that is collected and actually recycled. In the absence of public information, it is presumable that the post-consumer (End-of-life) CNT components go into the waste system and are landfilled, incinerated or recycled with other black plastics [17,18,19].

The management of polymer/CNT nanocomposite waste is a difficult task. As in the case of unfilled polymer waste, the methods such as waste burning, incineration and burying underground show a negative effect on the environment due to the formation of dust, fumes and toxic gases in the air or the pollution of the underground water [17], since the complete destruction of CNTs cannot be ensured [17]. The probability of CNTs surviving the combustion is strongly affected by the type of polymer matrix into which the CNTs are introduced and the combustion temperature, increasing with decreasing combustion temperature [17]. Therefore, it is preferable that the polymer/CNT products be recycled.

Mechanical recycling is currently the dominant, well-established method/technology of recycling the post-consumer thermoplastic waste in Europe [20,21] because it is a low-polluting process with low energy consumption and economic benefits in reusing rejected parts in the production of plastic waste [22,23,24]. On the other hand, mechanically recycled plastics are usually associated with poor-quality materials. In general, recycled materials show a loss of mechanical properties due to degradation processes occurring during processing, life cycle and mechanical recycling [20,21]. The degree of degradation of recycled plastic can vary from plastic to plastic, depending on the degradation processes associated with the polymer matrix, such as thermos-oxidative and shear-induced chain scission, chain branching or cross-linking [25,26,27,28,29], which in turn affect the processability and lower the mechanical properties.

Products made of thermoplastic polymer/CNT nanocomposites do not differ from thermoplastic waste when it comes to mechanical recycling. If a separate recycling stream for polymer/CNT nanocomposites exists, they can be easily mechanically recycled alongside their un-filled counterparts. However, contamination of plastics, collecting, sorting and degradation remain the major barriers to efficient recycling [20]. Also, mechanical recycling of polymer/CNT waste struggles to meet the challenging requirement of production at scale because of their low volume.

Although major efforts have been made within the area of polymer/CNT nanocomposites, including processing, characterization and manufacturing, so far, little focus has been directed to the area of recyclability and mechanical recycling of this class of materials.

Svensson et al. [30] studied the effect of multiple recycling on the thermal and mechanical behavior of the HDPE filled with 3 wt.% of CNTs and reported that no significant changes were found in up to ten cycles. Zhang et al. [25] found that the viscosity of the recycled PP filled with 3 wt.% of CNTs decreased with increasing recycling up to twenty cycles due to polymer chain degradation, while the mechanical properties exhibited in-significant changes. The PP/CNT nanocomposites showed an increase in toughness with recycling due to the changes in the crystallization behavior, while the presence of CNTs improved the recycling resistance. Zhang et al. [26] analyzed the effect of the recycling process on the PC filled with 3 wt.% of CNTs up to twenty cycles. The research showed no significant changes in the chemical structure of the recycled PC/CNT nanocomposite. The decrease in the rheological and mechanical properties with recycling was attributed to CNT breakage or chain scission and molecular weight reduction due to high shear rates and thermal stresses found in the multiple recycling cycles. Stan et al. [31] investigated the influence of reprocessing on the PP filled with 1, 3, and 5 wt.% of CNTs, and no significant changes were observed with respect to processability, electrical conductivity, elastic modulus and yield strength. However, it was found that the stress and strain at break increased after reprocessing, indicating the reduction of CNT aggregates during mechanical reprocessing. Stan et al. [32] studied the impact of mechanical recycling and remanufacturing by injection molding on the behavior of LDPE nanocomposites with 0.1–5 wt.% of CNTs. After the first recycling process, it was found that the mechanical, electrical and rheological properties of the recycled nanocomposites were similar to those of the virgin nanocomposites.

From the literature review [25,26,30,31,32], it can be concluded that knowledge of the process-induced degradation of the physical, mechanical and thermal properties of polymer/CNT nanocomposites due to recycling operations (grinding and melt reprocessing) is of paramount importance in order to understand the role of CNTs during the recycling process and to consider the use of the recycled nanocomposites for industrial applications.

The aim of this study is to investigate the recyclability of EVA/CNT nanocomposite waste. For this purpose, pre-consumer EVA/CNT nanocomposite waste with 1, 3 and 5 wt.% of multi-walled carbon nanotubes (MWCNTs) was recycled by mechanical grinding and reprocessed by injection molding up to three cycles, and the mechanical, thermal, electrical, and rheological properties of the recycled EVA/CNT nanocomposites were investigated after each reprocessing cycle and compared to those of virgin EVA/MWCNT nanocomposites—which are considered as the reference.

## 2. Research Methodology

**Figure 1:**
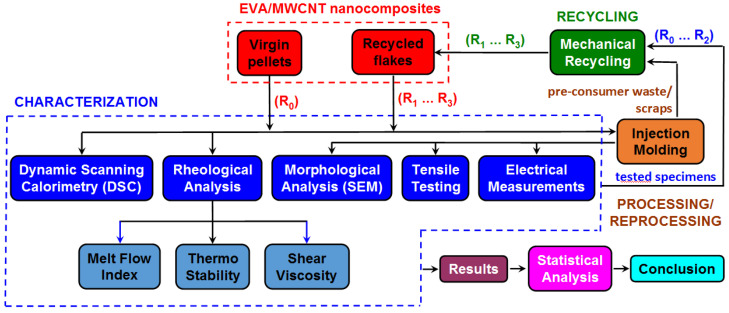
Research methodology.

Figure 1 presents the research methodology adopted in this paper to mimic thermo-mechanical recycling and to investigate the effect of reprocessing on the thermal, rheological and mechanical properties of EVA/MWCNT nanocomposites.

Primary mechanical recycling involves the recycling of pre-consumer waste and refers to grinding and reprocessing by injection molding. First, the virgin EVA/MWCNT nanocomposites (i.e., R_0_) were characterized based on differential scanning calorimetry (DSC) measurements, melt flow index (MFI) measurements, capillary viscosity measurements, scanning electron microscopy (SEM), mechanical tensile tests and electrical measurements. Then, for each MWCNT loading, the nanocomposite waste (i.e., injection molding waste and injection-molded samples after testing) was subjected to consecutive grinding into flakes and reprocessing by injection molding cycles (1 to 3 times), and the effect of recycling on thermal, rheological, electrical and mechanical properties of recycled nanocomposites was investigated using the same characterization methods. The experimental data were analyzed based on the main effect plot and analysis of variance.

## 3. Materials and Methods

### 3.1. Virgin Materials

In this study, an ethylene-vinyl acetate (EVA) copolymer matrix was selected due to its outstanding properties such as high tensile strength, friction coefficient, thermal resistance, endurance at both low and moderate temperatures, transparency, flexibility, and UV radiation resistance [9,33,34,35,36]. This copolymer is also noted by low water absorption, temperature toughness, cross-linking temperature, or resin cost. In addition, the EVA copolymer stands out due to its good processability by injection molding [9,33,34,35,36].

This study involves the recycling of virgin EVA/MWCNT nanocomposites that were purchased from Nanocyl (EVA, Nanocyl^TM^, Sambreville, Belgium) in the form of pellets. These nanocomposites are based on ethylene-vinyl acetate copolymer with 20% vinyl acetate content (EVA, type Alcudia^®^ PA-420, Repsol, Madrid, Spain [37]) and 1, 3, and 5 wt.% of MWCNTs (NC7000, Nanocyl^TM^, Sambreville, Belgium). The reported characteristics of NC7000 MWCNTs are as follows: average nanotube diameter of 9.5 nm, average nanotube length of 1.5 μm, carbon purity > 90%, surface area 250–300 m^2^/g, volume resistivity 10^−4^ Ω cm and metal oxide 10% [38]. The virgin nanocomposites were obtained by directly melt mixing the EVA with 1, 3 and 5 wt.% of MWCNTs at 140 °C using a twin-screw extruder with an L/D length of 48.

### 3.2. Mechanical Recycling of EVA/MWCNT Nanocomposites

Mechanical recycling of the EVA/MWCNT nanocomposites was simulated by consecutive grinding and reprocessing by injection molding. In the absence of post-consumer waste, the pre-consumer (post-industrial) EVA/MWCNT nanocomposite waste that consists of injection molding waste and injection-molded samples after characterization was considered. It should be noted that the recycled nanocomposite waste comes from clean laboratory waste, which was collected and stored in a clean laboratory environment without contaminants; therefore, the washing process was not considered.

The EVA/MWCNT nanocomposite waste, sorted by MWCNT loading, was ground into flakes with sizes from 2 to 5 mm using a roller shredder machine (GS 17/22, Dega Plastics, Brescia, Italy). The nanocomposite flakes were then used directly to produce new samples by injection molding.

The mechanical recycling was repeated three times, and each cycle (grinding and injection molding) was labeled as R_1_, R_2_ and R_3_. The virgin nanocomposites (both pellets and injection-molded samples) were labeled as R_0_.

**Figure 2:**
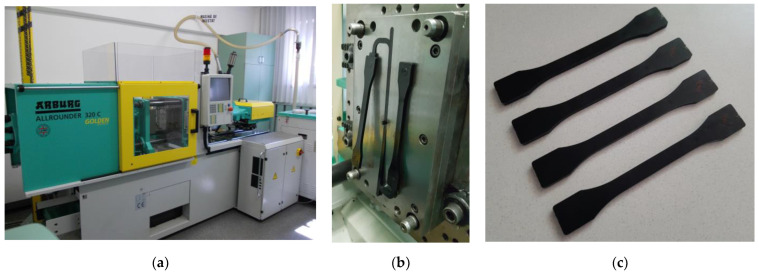
Injection molding process: (**a**) injection molding machine, (**b**) two-cavity mold, and (**c**) injection-molded tensile specimens.

Tensile specimens according to ISO 527 standard (type 1B) [39] with a thickness of 4 mm were injection-molded using an injection molding machine (Allrounder 320 C 500-170, Arburg, Loßburg, Germany) equipped with a two-cavity mold (Figure 2). Both virgin and recycled nanocomposites were dried for 2 h at 80 °C using a granulate dryer (Thermolift 100-2, Arburg, Loßburg, Germany) before each reprocessing cycle. The tensile specimens were conditioned in a standard laboratory environment for 24 h before testing.

The injection molding process was performed based on a full factorial three-level design with two factors (see Table S1 of Supplementary Material), namely melt temperature (140, 160, and 180 °C) and MWCNT loading (1, 3, and 5 wt.%). The remaining process parameters were set as follows: injection molding pressure of 800 bar, holding pressure of 90% injection pressure, a flow rate of 15 cm^3^/s; holding time of 10 s; cooling time of 20 s, and a mold temperature of 30 °C.

### 3.3. Characterization of the EVA/MWCNT Nanocomposites

#### 3.3.1. Differential Scanning Calorimetry

Differential scanning calorimetry (DSC) was conducted to investigate the effect of thermo-mechanical recycling on the thermal behavior of EVA/MWCNT nanocomposites. A DSC equipment (DSC 200 F3 Maia, Netzsch, Selb, Germany) was used to carry out non-isothermal DSC analysis after each reprocessing cycle. Samples of about 25 mg were sealed into a 100 µL aluminum pan and tested for two endothermic scans between −100 and 150 °C and one exothermic scan between 150 and −100 °C at a scanning rate of 10 °C/min. Before the endothermic and exothermic scans, the samples were held for 3 min at temperatures of −100 and 150 °C, respectively. A nitrogen flow rate of 50 mL/min was used, and a scanning rate of 10 °C/min. The glass transition temperature was extracted using the standard DSC analysis routine—midpoint option on the second heating curve.

The crystalline content ($\chi_{c}$) was calculated using Equation (1) [11,40]

(1) $$\chi_{c}=\frac{1}{1-wt.\%}\cdot \frac{\Delta H_{m}}{\Delta H_{m}^{0}}\times 100(\%),$$

in which $\Delta H_{m}$ is the melt enthalpy of the nanocomposite (J/g), $\Delta H_{m}^{0}$ is the melt enthalpy of 100% crystalline polymer, and *wt.%* is the CNT weight fraction. The melting enthalpy of 100% PE was taken as 286.3 J/g.

#### 3.3.2. Rheological Analysis

The melt flow index (MFI) was measured using a melt indexer (Melt Flow Quick Index 7021-7022, Instron, Norwood, MA, USA) at 190 °C using a load of 2.16 kg, according to the ISO 1133 standard [41]. For each wt.% of MWCNTs, the MFI test was repeated five times in order to quantify the variability of the measurements.

The rheological behavior of both virgin and recycled nanocomposites was assessed in shear flow using a capillary rheometer (RG75, Göttfert, Buchen, Germany) equipped with a capillary die with a length-to-diameter ratio (L/D) of 30:1. The viscosity was measured at 140, 160 and 180 °C and shear rates ranging from 100 to 5000 s^−1^, which cover most polymer manufacturing processes, including 3D printing, extrusion, and injection molding. The initial melting time was set to 10 min. It should be notated that before testing, the nanocomposites in the form of pellets and flakes were vacuum dried at 60 °C for 3 h using a vacuum drying oven (EV-50, Raypa, Barcelona, Spain).

The thermal-rheological stability of the EVA/MWCNT nanocomposites was characterized using the same capillary rheometer equipped with a capillary die with an L/D of 20:1. The flow behavior was monitored over time at 180 °C and 200 s^−1^ shear rate.

#### 3.3.3. Morphological Analysis

The morphology of the EVA nanocomposite, including the influence of reprocessing on the dispersion of MWCNTs within the EVA matrix, was analyzed by scanning electron microscopy (SEM). Injection molded specimens of each EVA nanocomposite were cryo-fractured using liquid nitrogen, and the core region of the fractured surface was analyzed using an electronic microscope (Quanta 200 3D, Fei, Hillsboro, OR, USA) at an acceleration voltage of 20 kV. The samples were sputter-coated with 5 nm of gold before analysis.

#### 3.3.4. Tensile Testing

The mechanical properties (e.g., Young’s modulus, maximum tensile strength, and stress and strain at break) were measured by tensile tests according to ISO 527 [39] using an universal testing machine (M350-5AT, Testometric, Rochdale, UK) at different crosshead speeds (5, 50 and 100 mm/min). The initial distance between the grips was 115 mm. At least five tests were performed in order to calculate the mean and the standard deviation of the mechanical properties.

#### 3.3.5. Electrical Measurements

The electrical resistance of the recycled EVA nanocomposite was measured using a DC power source (B2961A, Keysight Technologies, Santa Rosa, CA, USA) at a voltage level of +/−200V using a direct current two-probe method. The electrical resistance, *R* (Ω), and conductivity (i.e., the inverse of the volume resistivity), *σ* (S/m), of EVA/MWCNTs were computed by Equation (2), respectively Equation (3) [14].

(2) $$R=2\cdot \frac{R_{+}\cdot R_{-}}{\left(R_{+}+R_{-}\right)}(Ω),\;$$

(3) $$\sigma ={\left(R\cdot \frac{S}{L}\right)}^{-1}(S\cdot m),\;$$

where: $R_{+}$ and $R_{-}$ are the average equivalent resistance of the circuit under positive and negative voltage, respectively; *S* is the cross-section area on the sample perpendicular to the current flow (i.e., 4 mm × 5 mm); *L* is the distance between the electrodes (i.e., 50 mm). It should be noted that at least five fresh specimens were tested for each combination of the testing parameters (e.g., recycling and MWCNT loading). To improve the contact between the samples and the electrodes, the contact surface was coated with graphite conductive paste.

#### 3.3.6. Statistical Analysis

The analysis of variance (ANOVA) and main effect plot were used to assess the influence of different factors and to quantify the relative contribution of those factors on the rheological, electrical and mechanical properties. It should be noted that the analysis of variance was performed after checking the data normality with the Anderson-Darling test and for variance homogeneity with Levene’s test. The statistical analysis was performed by Minitab (16, Minitab LLC, State College, PA, USA) at a significance level of α=0.05. The effect of a factor is significant if p−value≤α [42].

## 4. Results and Discussion

### 4.1. Thermal Behavior

**Figure 3:**
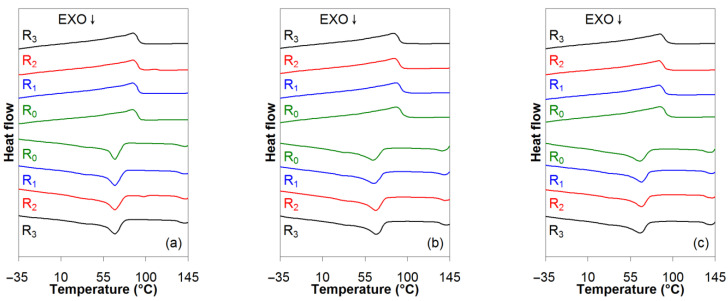
Effect of reprocessing cycle on the first cooling and second heating scans of the EVA/MWCNT nanocomposites with (**a**) 1 wt.%, (**b**) 3 wt.%, and (**c**) 5 wt.% of MWCNTs.

The DSC curves of the EVA/MWCNT nanocomposites corresponding to the first cooling and second heating scans are shown in Figure 3. It should be noted that the DSC scans for the R_1_, R_2_ and R_3_ cycles are shifted with respect to R_0_ for clarity only.

Table 1 presents the glass transition (*T_g_*), crystallization (*T_c_*), melting temperatures (*T_m_*), enthalpy (*ΔHm*, *ΔHc*) and crystallinity ($\chi_{c}$) for both virgin and recycled nanocomposites. For all nanocomposites, the crystallization temperatures were approximately 65 to 67 °C, the melting temperature was 85 to 88 °C, and the glass transition temperatures were −24 to −31 °C. Although there are some changes of 1 or 2 °C in the thermal properties of EVA/MWCNT nanocomposites with respect to recycling or MWCNT loading (Table 1), overall, statistical analysis of the DSC results indicates that neither mechanical recycling nor MWCNT loading has no significant effect on these temperatures. This behavior can be attributed to a very good interaction between the EVA matrix and nanotubes.

The results of the DSC analysis were used to assess the process window, defined as the difference between the melt and crystallization temperatures, *T_m_*–*T_c_*, and the effect of MWCNTs and recycling on the process window. As shown in Table 1, the processing window varies from 18 to 24 °C, with multiple samples in the same processing window. Therefore, it can be concluded that the processing window is not significantly affected by either the addition of MWCNTs or the recycling cycle.

The crystallinity varied from approximately 20% to 26%. For a given MWCNT loading, the value $\chi_{c}$ decreases with the increase of recycling numbers, and the effect is more important at lower MWCNT loading. After three reprocessing cycles, the degree of crystallinity decreased by 14%, 17% and 8% for 1 wt.%, 3 wt.% and 5 wt.%, respectively. The degree of crystallinity decreased with increasing nanotube loading, and the influence of nanotubes on the crystallinity is more important for the virgin nanocomposite (about 13% decrease with increasing nanotube loading from 1 to 5 wt.%). Overall, statistical analysis indicates that both mechanical recycling (*p*-value = 0.012) and MWCNT loading (*p*-value = 0.01) has a significant effect on crystallinity after three reprocessing cycles. Since crystallinity is related to molecular weight reduction [43,44,45], it can be suggested that molecular weight reductions were sufficient to produce major changes in crystallinity.

### 4.2. Melt Flow Index

Generally, the MFI of recycled polymers is found to increase with reprocessing cycle, which is attributed to the thermal degradation of the polymer due to the increase in the residence time [46,47,48,49,50,51,52] and over-drying [30,51,53,54] or excess moisture [27,55]. Multiple mechanical recycling and reprocessing of the thermoplastic/CNT nanocomposites can affect the CNT-CNT and polymer-CNT interactions [56,57,58,59,60,61,62] due to multiple exposures to high temperatures and shear rates causing cross-linking, scission or branching of polymer chains [25,26,27,28,29].

**Figure 4:**
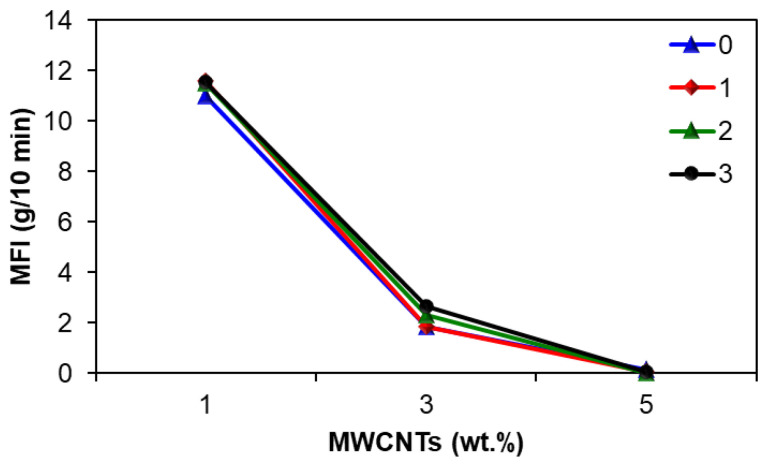
Effect of recycling on the MFI of EVA/MWCNT nanocomposites.

The MFI of the virgin and recycled EVA/MWCNT nanocomposites are presented in Figure 4. It should be noted that the standard deviation was less than or equal to 0.4, 0.1, and 0.01 for 1. 3 and 5 wt.% MWCNTs, respectively. A high value indicates lower viscosity and better processability. The MFI of virgin EVA copolymer was reported as 20 g/10 min (190 °C, 2.16 kg) [63].

The results of MFI indicate that the addition of MWCNTs significantly decreases the MFI of virgin nanocomposites as compared to the EVA copolymer. For example, the MFI of the virgin EVA/MWCNT nanocomposites decreased from 10.98 to 0.13 g/10 min (190 °C) when the MWCNT loading increased from 1 to 5 wt.%, and a similar trend is found for each recycling cycle. It may be due to the formation of strong intermolecular bonds between the EVA matrix and nanotubes, which ultimately imposes difficulties in melting the nanocomposites and hence leads to a decrease in MFI [64]. The presence of MWCNTs affects the MFI of nanocomposites through chain mobility. The addition of MWCNTs blocks the movement of the polymer chains during flow, resulting in a significant increase in the flow resistance [31,32].

On the other hand, it can be seen that the MFI increased only slightly after the first mechanical recycling cycle and then remained constant as the number of recycling increased further. It may be due to the protective effect of MWCNTs during mechanical recycling, especially on the chain scission, which is the main degradation mechanism [65].

Since the MFI provides an indirect assessment of the molecular weight based on the flowability at relatively low shear rates [66,67,68], the relative melt flow index was computed as the ratio between the MFI of the virgin and recycled nanocomposite. After three reprocessing cycles, no significant changes were observed in the relative MFI of the recycled nanocomposites with 1 wt.% and 5 wt.%. However, for the nanocomposite with 3 wt.%, the relative MFI increased from 1.01 to 1.42 after three reprocessing cycles, indicating possible degradation of the EVA matrix.

As can be seen in Table 1 and Figure 4, there is a general correlation between the MFI and melting enthalpy as the melting enthalpy decreases with decreasing MFI due to increasing CNT loading. However, a stronger correlation was found between the MFI and the crystallization properties. The crystallization peak temperature and the enthalpy of crystallization decrease in order to decrease MFI.

The statistical analysis of the MFI indicated that the recycling does not significantly affect the MFI of EVA/MWCNT nanocomposite (*p*-value = 0.313), while the effect of MWCNT loading is statistically significant (*p*-value = 0), and the effect associated with nanotube loading is negative.

### 4.3. Rheological Behavior

**Figure 5:**
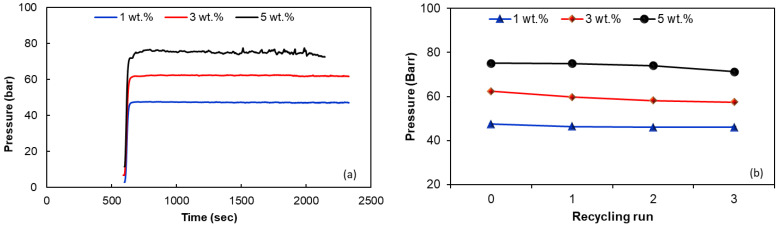
Thermo-stability curves for the virgin EVA/MWCNT nanocomposites (**a**) and the effect of recycling run on the melt pressure during capillary extrusion at 200 s^−1^ and melt temperature of 180 °C (**b**).

Monitoring the variation of pressure (or melt shear viscosity) over time provides information on the stability of the nanocomposite during melt extrusion processes. Figure 5a shows the thermos-stability curves for the virgin EVA/MWCNT nanocomposites during capillary extrusion at a constant shear rate of 200 s^−1^ and melt temperature of 180 °C. The nanocomposites exhibit good flow stability during extrusion with no significant degradation over time (pressure drop), regardless of MWCNT loading. It is also noted that the addition of MWCNTs into the EVA matrix significantly increased the bearing capacity of the molten nanocomposites [51,56,57,69,70]. For example, the average pressure increased by 37% when the MWCNT loading increased from 1 to 5 wt.% of MWCNTs.

Figure 5b shows the effect of mechanical recycling and reprocessing on the pressure during capillary extrusion of EVA/MWCNT nanocomposites. It should be noted that the pressure represents the mean value over 1200 s of extrusion flow at a constant shear rate of 200 s^−1^ and melt temperature of 180 °C. Figure 5b reveals that the pressure slightly decreased with increasing reprocessing cycle. However, the influence of reprocessing cycle on the thermo-stability of the EVA/MWCNT nanocomposites is negligible, with less than a 5% decrease.

**Figure 6:**
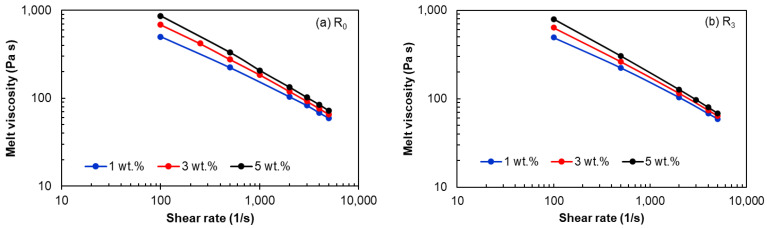
Melt shear viscosity as a function of shear rates and MWCNT wt.% at 160 °C: (**a**) R_0_ and (**b**) R_3_.

The apparent log-log flow curves (i.e., shear viscosity vs. shear rate) for the R_0_ and R_3_ recycled EVA/MWCNT nanocomposites are shown in Figure 6 for the melt temperature of 160 °C. It can be seen that the behavior of the recycled nanocomposites is similar to that of virgin nanocomposites, indicating that the processability of the recycled nanocomposites is not significantly affected by mechanical recycling. The flow curves show the familiar pattern of increasing melt viscosity as the MWCNT content increases and a typical shear-tinning behavior over the range of studied shear rates.

**Figure 7:**
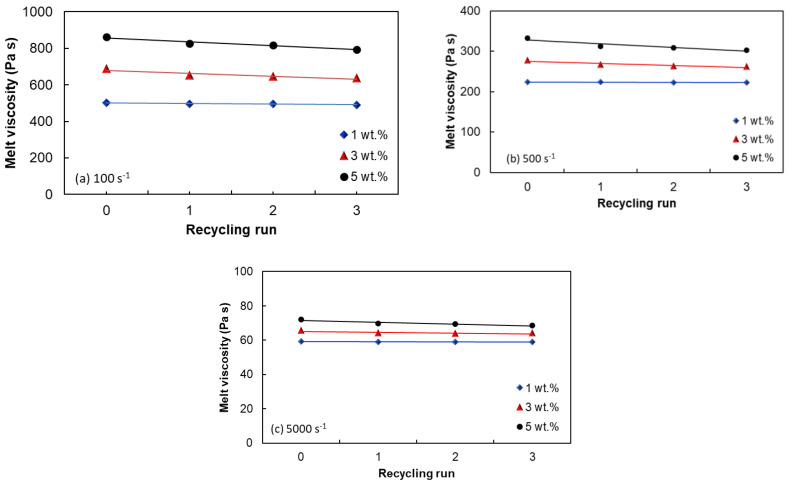
Effect of shear rate and recycling run on the melt shear viscosity of EVA/MWCNT nanocomposites: (**a**) 100 s^−1^, (**b**) 500 s^−1^ and (**c**) 5000 s^−1^.

The effect of recycling the melt shear viscosity at different shear rates is depicted in Figure 7. At low shear rates, the first graph in Figure 7 shows a slight reduction of the shear viscosity after 3 cycles for the nanocomposites with 3 wt.% and 5 wt.%. This can happen as a result of thermomechanical degradation of the polymer matrix, chain scissions of the macromolecules of the polymer matrix or CNT breakage due to shear forces [7,20,69,71]. Figure 7b,c, however, shows a steady shear viscosity at higher shear rates.

**Figure 8:**
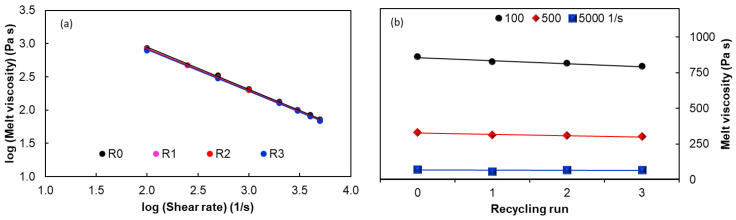
Melt viscosity vs. (**a**) shear rate and (**b**) recycling run of the EVA/MWCNT nanocomposite with 5 wt.% (160 °C).

Figure 8a shows the effect of the recycling on the flow curves for EVA/MWCNT nanocomposite with 5 wt.% at 160 °C. It can be seen that the flow curves (before and after the mechanical recycling) are linear and almost overlapping. A closer look at the variation of melt shear viscosity with shear rate and recycling run in Figure 8b indicates that at low shear rates, the melt viscosity decreases with increasing recycling run (about 10–15%), whereas at higher shear rates, the effect of recycling is only marginal (less than 5 %). This finding is very important from a practical point of view. In general, the reduction of the melt viscosity requires the recalibration of the processing equipment. However, most of the manufacturing processes (injection molding, extrusion) operate at medium and high shear rates, and therefore the processing window for the recycled EVA/MWCNT nanocomposites is not affected.

The reduction in the melt shear viscosity is greater at low shear rates. The reduction of the melt viscosity requires the recalibration of the processing equipment. However, most of the manufacturing processes (injection molding, extrusion) operate at medium and high shear rates, and therefore the processing window is not affected.

Both virgin and recycled nanocomposites obey the power law in the range of studied shear rates [72,73].

(4) $$\eta_{a}=K\cdot {\dot{\gamma }}_{a}^{n-1}\;(\mathrm{Pa}\cdot s),$$

where $\eta_{a}$ is the apparent shear viscosity, ${\dot{\gamma }}_{a}$ is the apparent shear rate, *K* is the consistency index, and *n* is the flow behavior index. The values of *K* and *n* are listed in Table 2 for different MWCNT loadings, temperatures, and reprocessing cycles.

As can be seen in Table 2, all nanocomposites exhibit shear-thinning behavior—ex-pressed as the exponent of the power law—with a flow index of 0.34–0.49 that hardly chances with recycling run.

### 4.4. Activation Energy

The activation energy of the polymer/MWCNT nanocomposite melts an important parameter for the melt manufacturing processes since it reflects the temperature sensitivity of the viscosity as higher activation flow energy leads to higher sensitivity of the nanocomposite to temperature [74]. In general, a reduction of the flow activation energy corresponds with a reduced influence of the temperature on the viscosity due to a pronounced interaction of the nanotubes with the polymer [9,56,57,58,59,60,61,62].

In this study, the flow activation energy was obtained based on the Arrhenius equation [9,32,75]

(5) $$\eta_{a}=A\cdot \exp\frac{E_{a}}{R\cdot T}$$

where $\eta_{a}$ is the apparent shear viscosity (Pa·s), *A* is a constant, $E_{a}$ is the flow activation energy (kJ/mol), *R* is gas constant (*R* = 8.314 J mol^−1^ K^−1^), and *T* is the absolute temperature (K).

**Figure 9:**
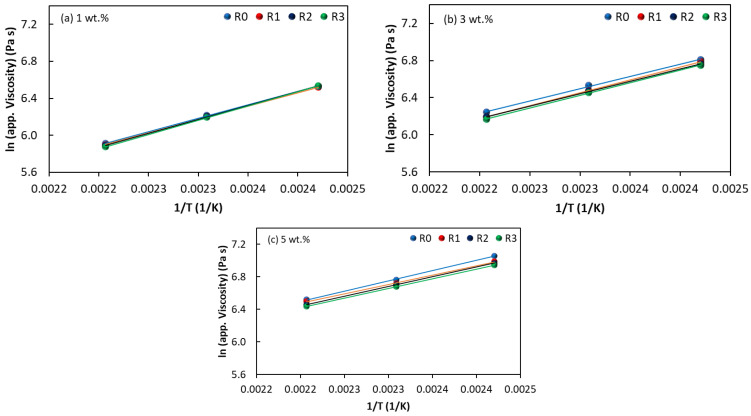
Influence of reprocessing on shear stress as a function of shear rate for the composites with (**a**) 1 wt.%, (**b**) 3 wt.% and (**c**) 5 wt.% of MWCNTs.

Figure 9 shows typical Arrhenius plots, e.g., *ln* shear viscosity (Pa s) vs. *ln* reciprocal absolute temperature (K^−1^) for the EVA/MWCNT nanocomposite as a function of recycling runs and nanotube loadings. It can be observed that the results obey the Arrhenius model very well, as the graphs indicate a linear trend of apparent melt shear viscosity vs. 1/T. Nearly identical parallel lines were obtained for the Arrhenius plots, which indicate similar apparent activation energy values after three cycles of recycling. For example, at a fixed shear rate of 100 s^−1^ and 5 wt.% of MWCNTs, a flow activation energy of 21 kJ/mol and 19.7 kJ/mol was estimated for the R_0_ and R_3_ nanocomposite, respectively.

**Figure 10:**
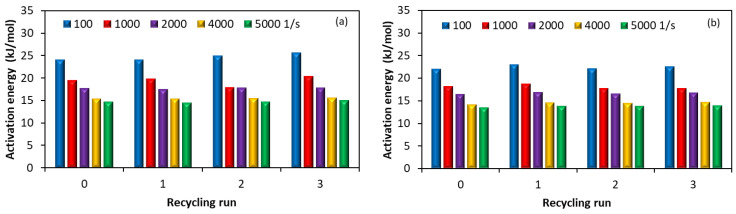

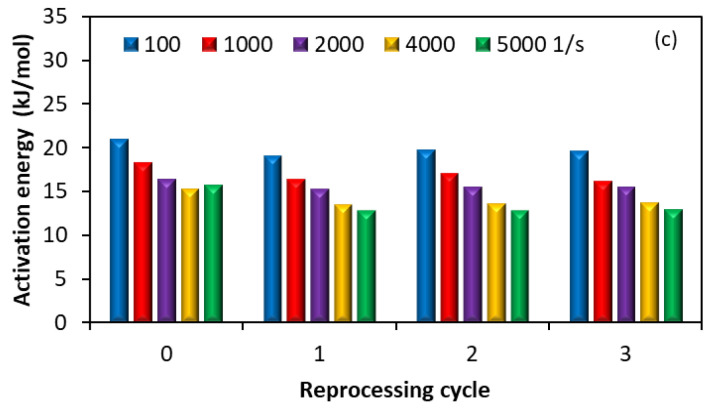
Influence of recycling and shear rate on the activation energy for the nanocomposites with (**a**) 1 wt.%, (**b)** 3 wt.% and (**c**) 5 wt.% of MWCNTs.

Figure 10 shows the values of the apparent activation energy as a function of reprocessing cycle and shear rates. It can be seen that the flow activation energy tends to decrease with increasing shear rates and with the addition of MWCNTs, which indicates that the sensitivity of viscosity to temperature reduces. Moreover, the effect of the shear rate is more important than the effect of MWCNT loading. The effect of increasing the melt shear viscosity from 100 to 5000 s^−1^ is a decrease of somewhere between 25 and 40% in activation energy, depending on the MWCNT loading. Furthermore, the effect of increasing the MWCNT loading from 1 wt.% to 5 wt.% is a decrease of about 15% in the activation energy. The flow activation energy decreases with increasing shear rate due to the fact that the molecules align in the flow direction [76,77], and this is supported by the melt flow index (Figure 4). The low viscosity sensitivity to temperature indicates that the recycled nanocomposites have the same broad processing temperature window as the virgin nanocomposites, simplifying the selection of processing temperature.

### 4.5. Morphological Analysis

**Figure 11:**
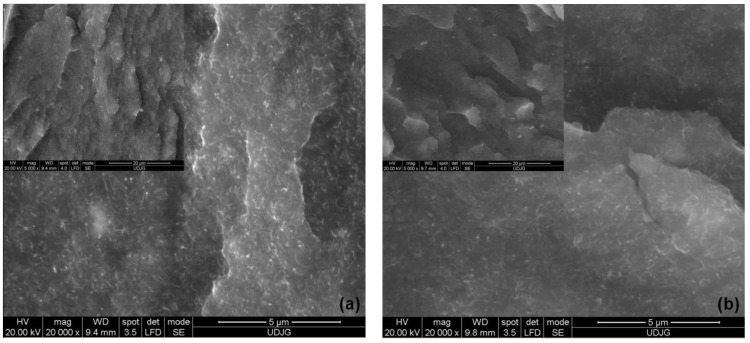

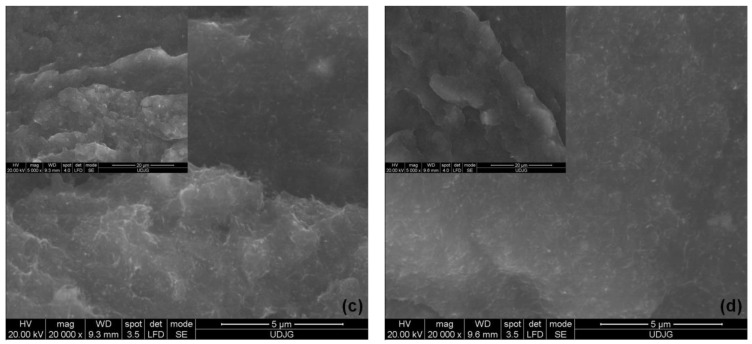
SEM micrographs of cryogenic surface fracture at 20,000× magnification (insets at 5000×) for the (**a**) R_0_, (**b**) R_1_, (**c**) R_2_ and (**d**) R_3_ composites with 5 wt.% of MWCNTs.

Figure 11 shows the SEM micrographs for the EVA/MWCNT nanocomposite with 5 wt.% of MWCNTs. It should be noted that the micrographs correspond to the core area of the injection-molded samples at 160 °C, perpendicular to the polymer flow direction. The SEM micrographs suggest that the adhesion between the polymer matrix and carbon nanotubes was not affected by the grinding process even after three reprocessing cycles, as only a few nanotubes were pulled out from the matrix. In general, as reported in the literature, higher carbon nanotube loading leads to the formation of unavoidable CNT agglomerates and clusters, which impact the mechanical and electrical properties of the nanocomposites [7,9,11,20,21,69,71,78]. However, as in Figure 11, the CNTs appear to be homogeneously dispersed by the shear flow during the injection molding process without visible local agglomerates. Moreover, it is reasonable to expect that any CNT agglomeration or cluster formed during the injection molding process are breakdowns during the mechanical recycling process [7,25,31,79].

### 4.6. Mechanical Properties

**Figure 12:**
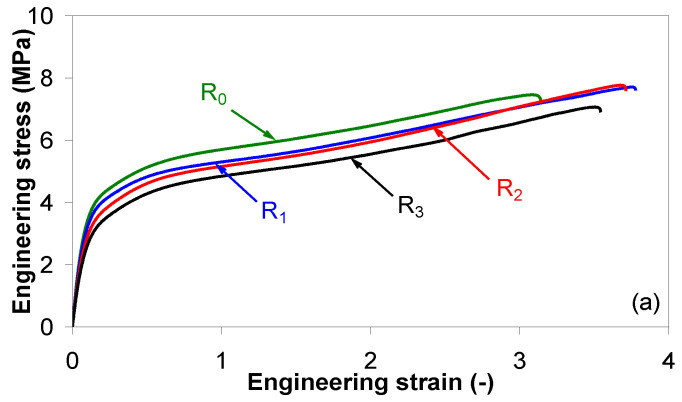

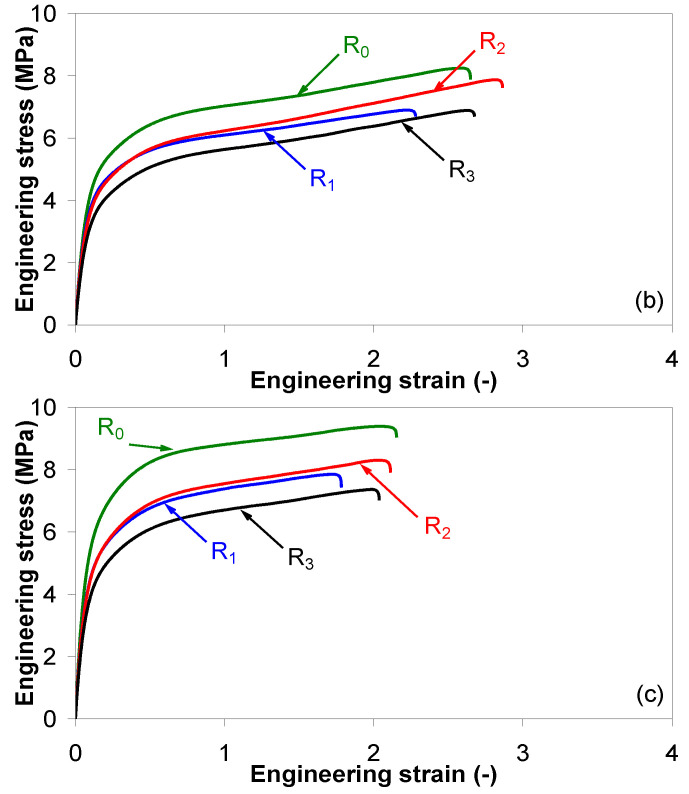
Effect of mechanical recycling on the stress-strain curves of the EVA/MWCNT nanocomposites with (**a**) 1 wt.%, (**b**) 3 wt.% and (**c**) 5 wt.% of MWCNTs at a crosshead displacement of 100 mm/min.

**Figure 13:**
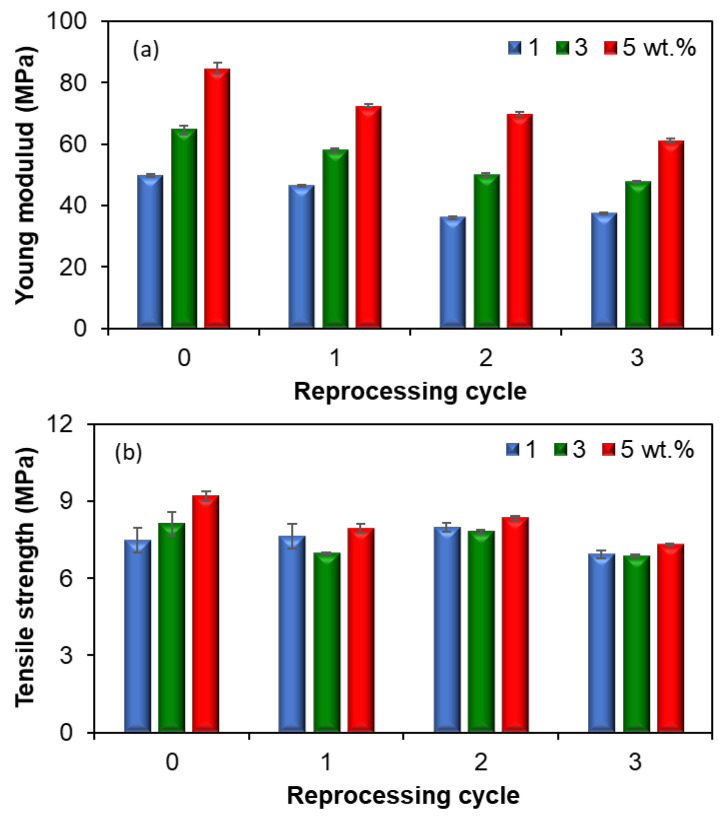

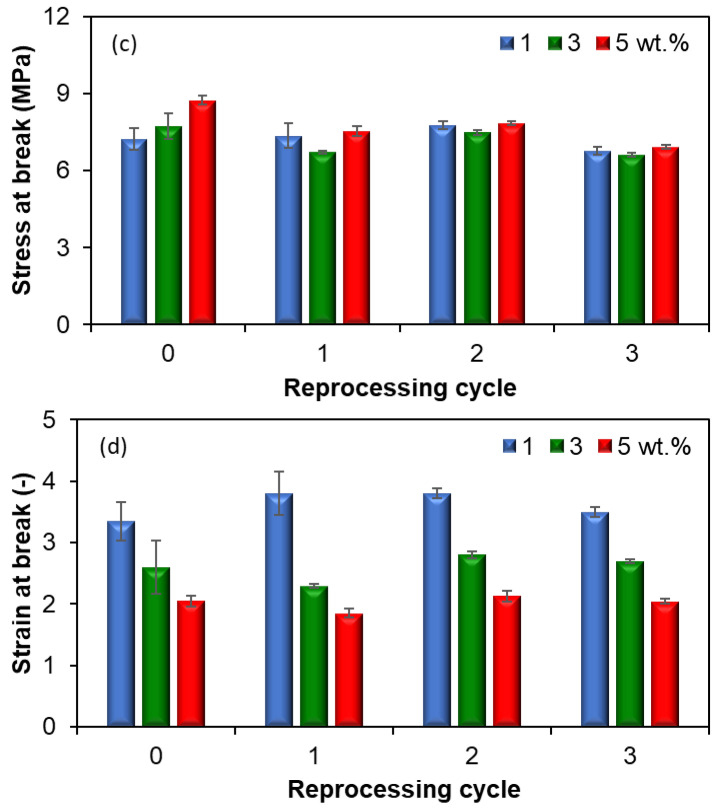
Effect of reprocessing cycle on the (**a**) Young’s modulus, (**b**) tensile strength, (**c**) stress at break and (**d**) strain at break for the EVA/MWCNT nanocomposites.

Representative stress-strain curves of EVA/MWCNT nanocomposites as a function of recycling run at a crosshead displacement of 100 mm/min are shown in Figure 12 for injection-molded samples at 160 °C. The corresponding mechanical properties (e.g., Young’s modulus, tensile strength, stress at break and strain at break) are presented in Figure 13. Overall, the stress level of the EVA/MWCNT nanocomposite decreases with reprocessing cycle and increases with increasing MWCNT loading. On the other hand, the ductility decreases due to the addition of MWCNTs. This is illustrated by the decrease in the elongation at break from about 350% at 1 wt.% MWCNTs to about 200% at 5 wt.% MWCNTs.

**Figure 14:**
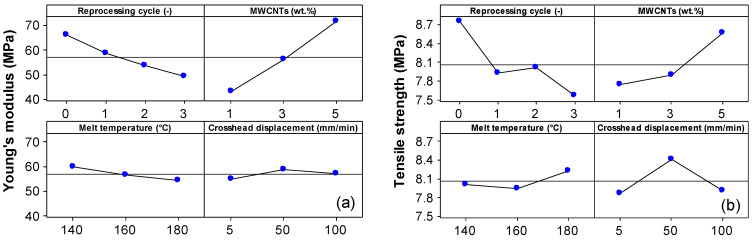

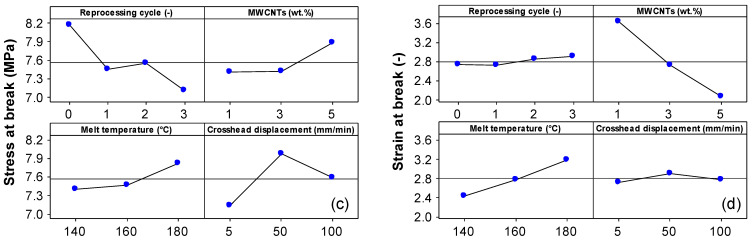
Main effect plot for the (**a**) Young’s modulus, (**b**) tensile strength, (**c**) stress at break and (**d**) strain at break for the EVA/MWCNT nanocomposites.

The effect of the reprocessing cycle, MWCNT wt.%, melt temperature, and crosshead displacement on the mechanical properties is illustrated in the main effect plot in Figure 14. To better elucidate the effect of these factors on the mechanical properties, the experimental data were analyzed using the analysis of variance in Minitab and the results are reported in Tables S2–S5 of Supplementary Material. It should be noted that the non-significant effects have not yet pooled into an estimated error. All main factors have a significant effect on the mechanical properties of EVA/MWCNT nanocomposites (*p*-value < 0.05).

The main effect plot in Figure 14 and ANOVA (see Tables S2–S5 of Supplementary Materials) show that the reprocessing has a decreasing effect on Young’s modulus, tensile strength and stress at a break of about 25.6%, 13.5%, respectively 12.9% compared to the virgin EVA/MWCNT. Similar behavior has been previously reported for recycled polymer/CNT nanocomposites [31,32,79]. The decrease in the mechanical properties with reprocessing cycle, except for the strain at the break that increased by about 6.5%, may be associated with thermal and mechanical degradation of the polymer matrix due to multiple exposures to high temperatures and high shear rates during the injection molding process. On the other hand, the CNT lengths can also be reduced by the mechanical stress occurred during grinding [26]. The decrease in the mechanical properties of the EVA/MWCNT nanocomposite also might be associated with the decrease in crystallinity degree [25,31,32,79]. The composites showed a reduction of the crystallization degree with reprocessing that affects the growth and formation of crystals, which determined the mechanical properties to decrease.

Furthermore, as expected, the effect of increasing MWCNT loading from 1 wt.% to 5 wt.% is an increase in mechanical properties except for the strain at the break that decreases about 43% in all nanocomposites. The main effect plot for melt temperature in Figure 14 also shows that higher tensile strength, stress at break and strain at break are obtained when the melt temperature is at a high value (180 °C), but higher Young’s modulus values are obtained with the melt temperature at a low level (140 °C).

The crosshead displacement also has a significant effect (*p*-value = 0) on all mechanical properties, but the magnitude of the contribution on different mechanical properties is different (see C % in Tables S2–S5). Figure 14 shows that increasing crosshead speed from 5 mm/min to 50 mm/min yields higher mechanical properties, whereas a further increase in the crosshead speed leads to a decrease in mechanical properties.

### 4.7. Electrical Conductivity

**Figure 15:**
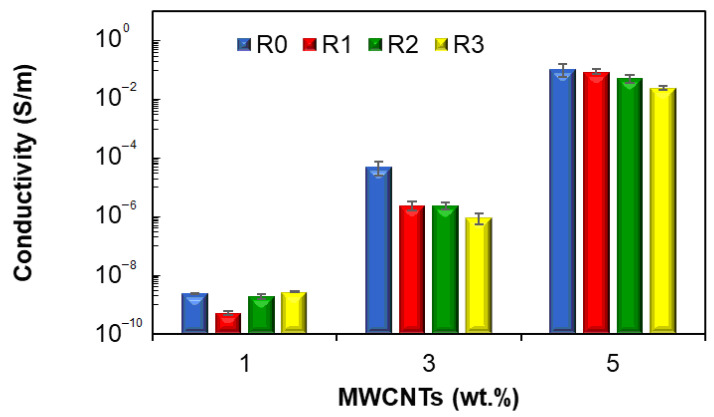
Electrical conductivity of EVA nanocomposites as a function of MWCNTs and recycling.

Figure 15 presents the effect of recycling and MWCNT loading on the electrical conductivity of EVA nanocomposites. It can be seen that for both virgin and recycled nanocomposites, the electrical conductivity increases with increasing MWCNT loading, spanning the insulating to the semi-conducting range. These results are in concordance with the reported literature on the recycling of polymer/CNT nanocomposites [31,32,79,80,81,82,83]. It can be seen that there is a stiff increase in the electrical conductivity at 3 wt.%, indicating that at this nanotube loading, conductive networks are fully developed, and the percolation threshold was achieved [82,84,85]. Regarding the effect of mechanical recycling electrical conductivity, Figure 15 indicates a decrease in the electrical conductivity with increasing recycling run. After three recycling cycles, the conductivity of the recycled nanocomposites (varying between about 10^−9^ S/m at 1 wt.% and 10^−2^ S/m at 5 wt.%) is about one order of magnitude lower as compared with the virgin nanocomposites (varying between about 10^−9^ S/m at 1 wt.% and 10^−1^ S/m at 5 wt.%), and the decrease is more important at 3 wt.%. The decrease in the electrical conductivity of the recycled nanocomposites can be explained by several mechanisms that occur during the mechanical recycling process, such as mechanical degradation due to grinding and injection molding [20,21,31,32,79] and CNT breakdown and deagglomeration under high shear rates during the injection molding [7,9,11,20,69,71,78]. These phenomena have a direct impact on the topology of the conductive network, i.e., smaller agglomerates have a decreasing effect on the electrical conductivity [84,86,87,88,89]. However, the conductivity of the recycled EVA nanocomposite with 5 wt.% MWCNTs are still in the semi-conducting range, indicating that these nanocomposites can be used in applications for which the primary requirement is related to semi-conducting properties or antistatic packaging. Statistical analysis of the experimental data shows that the MWCNT loading has a significant influence on the conductivity (*p*-value = 0.005), while the effect of recycling is not statistically significant (*p*-value = 0.454).

## 5. Conclusions

In this study, the effect of mechanical recycling on thermal, rheological and mechanical properties of ethylene-vinyl acetate/multi-walled carbon nanotube (EVA/MWCNT) nanocomposites was investigated. To mimic the thermo-mechanical recycling process, virgin EVA filled with 1, 3 and 5 wt.% of MWCNTs was subjected to consecutive grinding and reprocessing by injection molding up to three cycles, and the changes induced by mechanical recycling were monitored by DSC, MFI, capillary rheology, SEM, electrical resistivity and tensile testing.

Based on the experimental results, it can be concluded that, with proper control over processing conditions, EVA/MWCNT nanocomposites can undergo at least three cycles of mechanical recycling without concern for significant loss of thermal and rheological performances and recalibration of the processing window. The deterioration of the mechanical properties is the primary challenge for the efficient mechanical recycling of EVA/MWCNT nanocomposites. The EVA/MWCNT nanocomposites retain close to 75% of the original elastic modulus after three recycling cycles and about 80–90% in the tensile strength, depending on the MWCNT loading—the effects are more severe at higher CNT loading. However, with proper control of the injection molding temperature and CNT loading, the mechanical properties can be tuned close to those of the virgin nanocomposites. The electrical conductivity of the recycled EVA/MWCNT nanocomposites is about one order of magnitude lower than that of the virgin nanocomposites, spanning the insulating to the semi-conducting range, depending on the CNT loading.

This work demonstrates the positive effect of CNTs on the processing window and end-user properties of mechanically recycled EVA/CNT nanocomposites. In particular, the recycled EVA/MWCNT nanocomposites could be used for electrostatic discharge (ESD) protective packaging where a low level of conductivity is enough, automotive parts that require electrostatic dissipation, or strain-based sensors that require a high level of conductivity coupled with good flexibility. Future work will address in-depth the influence of reprocessing on the molecular weight distribution and crystallinity of the polymer/CNT nanocomposites. In addition, since in this paper, the clean waste selected based on the CNT loading was considered, future work will address the recycling of post-consumer polymer/CNT nanocomposites.

## Figures and Tables

**Table 1:** DSC properties of EVA/MWCNT nanocomposites.

MWCNTs (wt.%)	Recycling Cycle	Cooling		Second Heating				*T_m_*–*T_c_* (°C)
		*T_c_* (°C)	*ΔH_c_* (J/g)	*T_g_* (°C)	*T_m_* (°C)	*ΔH_m_* (J/g)	$\chi_{c}\;(\%)$	
1	R_0_	67	70.72	−25	86	70.13	24.74	19
	R_1_	67	68.39	−24	86	65.01	22.94	19
	R_2_	67	63.18	−28	86	57.57	20.31	20
	R_3_	67	60.74	−27	86	62.83	22.17	20
3	R_0_	64	71.85	−24	88	76.51	27.55	24
	R_1_	64	64.16	−27	88	64.24	23.13	24
	R_2_	66	64.41	−27	86	61.81	22.26	19
	R_3_	67	59.77	−30	86	59.97	21.59	19
5	R_0_	65	58.82	−29	87	65.47	24.07	22
	R_1_	66	59.59	−31	85	58.32	21.44	18
	R_2_	67	57.07	−28	85	56.91	20.92	18
	R_3_	65	54.34	−27	87	60.27	22.16	20

**Table 2:** The consistency and shear-thinning index for the EVA/MWCNT nanocomposites.

Temperature, (°C)	MWCNTs, (wt.%)	Consistency Index, *K* (Pa·s^n^)				Shear-Thinning Index, *n* (−)			
		Reprocessing Cycle, (−)							
		R_0_	R_1_	R_2_	R_3_	R_0_	R_1_	R_2_	R_3_
140	1	10,573	10,098	10,176	10,288	0.42	0.42	0.42	0.42
	3	17,121	16,461	15,496	15,122	0.37	0.37	0.38	0.38
	5	24,456	22,818	22,780	21,151	0.35	0.34	0.34	0.35
160	1	6314	6283	6310	6161	0.46	0.46	0.45	0.46
	3	11,536	10,177	9956	9719	0.40	0.41	0.41	0.41
	5	16,607	15,427	15,371	14,377	0.36	0.37	0.37	0.38
180	1	4181	4012	3969	3931	0.48	0.49	0.49	0.49
	3	7561	7496	6939	6515	0.43	0.42	0.43	0.44
	5	11,884	11,376	10,868	10,570	0.38	0.38	0.39	0.39

## Data Availability

The data presented in this study are available in an article or supplementary material.

## Supplementary Materials

The following are available online at https://www.mdpi.com/article/10.3390/polym15030583/s1, Table S1. Injection molding experimental plan, Table S2. ANOVA of Young’s modulus (MPa) for the EVA/MWCNT nanocomposites, Table S3. ANOVA of the tensile strength (MPa) for the EVA/MWCNT nanocomposites, Table S4. ANOVA of the stress at break (MPa) for the EVA/MWCNT nanocomposites, Table S5. ANOVA of the strain at break (-) for the EVA/MWCNT nanocomposites.
