# Supplementary material for: Mechanical Recycling of Ethylene-Vinyl Acetate/Carbon Nanotube Nanocomposites: Processing, Thermal, Rheological, Mechanical and Electrical Behavior
Source: Polymers (Basel). 2023 Jan 23;15(3):583. doi: 10.3390/polym15030583 (PMC9919012; doi:10.3390/polym15030583)
Supplement: Supplementary file 1 [file polymers-15-00583-s001.zip › polymers-2141642-supplementary.pdf]

Supplementary materials

Mechanical Recycling of Ethylene-Vinyl Acetate/Carbon Nanotube Nanocomposites: Processing, Thermal, Rheological, Mechanical and Electrical Behavior

Ionut-Laurentiu Sandu*, Felicia Stan and Catalin Fetecau*

Center of Excellence Polymer Processing, Dunarea de Jos University of Galati, 47 Domneasca, 800 008 Galati, Romania

* Correspondence: laurentiu.sandu@ugal.ro (I.-L.S.); catalin.fetecau@ugal.ro (C.F.).

1. Tables

Table S1. Injection molding experimental plan.

Experiment No.	Melt temperature, (°C)	MWCNT loading, (wt.%)
1	140	1
2	140	3
3	140	5
4	160	1
5	160	3
6	160	5
7	180	1
8	180	3
9	180	5

Table S2. ANOVA of Young's modulus (MPa) for the EVA/MWCNT nanocomposites.

Source	DOF	Seq. SS	Adj. MS	F-test	P-value	C, (%)
R_n, (-)	3	4264.51	1421.50	431.40	0	20.31
MWCNTs, (wt.%)	2	14756.09	7378.05	2239	0	70.29
T, (°C)	2	524.26	262.13	79.55	0	2.50
v, (mm/min)	2	279.69	139.85	42.44	0	1.33
R_n * *MWCNTs*	6	389.85	64.98	19.72	0	1.86
R_n * *T*	6	19.60	3.27	0.99	0.438	0.09
R_n * *v*	6	321.81	53.63	16.28	0	1.53
MWCNTs * *T*	4	149.03	37.26	11.31	0	0.71
MWCNTs * *v*	4	40.64	10.16	3.08	0.022	0.19
T * *v*	4	22.63	5.66	1.72	0.156	0.11
Error	68	224.07	3.30			1.07
Total	107	20992.19				100.00
		SD = 1.82; R^2 = 98.93%; R^2 (adj) = 98.32%.				

Table S3. ANOVA of the tensile strength (MPa) for the EVA/MWCNT nanocomposites.

Source	DOF	Seq. SS	Adj. MS	F-test	P-value	C, (%)
R_n, (-)	3	20.06	6.69	111.8	0	33.60
MWCNTs, (wt.%)	2	13.95	6.97	116.6	0	23.37
T, (°C)	2	1.53	0.76	12.76	0	2.56
v, (mm/min)	2	6.52	3.26	54.52	0	10.92
R_n * *MWCNTs*	6	4.04	0.67	11.27	0	6.77
R_n * *T*	6	0.38	0.06	1.05	0.401	0.63
R_n * *v*	6	2.78	0.46	7.74	0	4.66
MWCNTs * *T*	4	3.25	0.81	13.61	0	5.45
MWCNTs * *v*	4	1.39	0.35	5.8	0	2.32
T * *v*	4	1.74	0.43	7.26	0	2.91
Error	68	4.07	0.06			6.81
Total	107	59.7				100.00
		SD = 0.25; R^2 = 93.19%; R^2 (adj) = 89.28%.				

Table S4. ANOVA of the stress at break (MPa) for the EVA/MWCNT nanocomposites.

Source	DOF	Seq. SS	Adj. MS	F-test	P-value	C, (%)
R_n, (-)	3	15.70	5.23	85.27	0	30.45
MWCNTs, (wt.%)	2	5.28	2.64	43.06	0	10.25
T, (°C)	2	3.78	1.89	30.82	0	7.34
v, (mm/min)	2	12.68	6.34	103.40	0	24.61
R_n * *MWCNTs*	6	3.19	0.53	8.66	0	6.19
R_n * *T*	6	0.45	0.07	1.21	0.309	0.87
R_n * *v*	6	2.21	0.37	6.01	0	4.29
MWCNTs * *T*	4	2.67	0.67	10.87	0	5.18
MWCNTs * *v*	4	0.49	0.12	1.98	0.107	0.94
T * *v*	4	0.92	0.23	3.76	0.008	1.79
Error	68	4.17	0.06			8.09
Total	107	51.54				100.00
		SD = 0.25; R^2 = 91.90%; R^2 (adj) = 87.26%.				

Table S5. ANOVA of the strain at break (-) for the EVA/MWCNT nanocomposites.

Source	DOF	Seq. SS	Adj. MS	F-test	P-value	C, (%)
R_n, (-)	3	0.69	0.23	15.44	0	1.16
MWCNTs, (wt.%)	2	45.37	22.68	1513	0	75.7
T, (°C)	2	10.28	5.14	342.7	0	17.15
v, (mm/min)	2	0.65	0.32	21.56	0	1.08
R_n * *MWCNTs*	6	0.45	0.08	5.02	0	0.75
R_n * *T*	6	0.1	0.02	1.09	0.375	0.16
R_n * *v*	6	0.35	0.06	3.84	0.002	0.58
MWCNTs * *T*	4	0.44	0.11	7.28	0	0.73
MWCNTs * *v*	4	0.31	0.08	5.11	0.001	0.51
T * *v*	4	0.28	0.07	4.74	0.002	0.47
Error	68	1.02	0.02			1.7
Total	107	59.93				100.00
		SD = 0.12; R^2 = 98.30%; R^2 (adj) = 97.32%.				
